# Species-specific roles of the Notch ligands, receptors, and targets orchestrating the signaling landscape of the segmentation clock

**DOI:** 10.3389/fcell.2023.1327227

**Published:** 2024-01-29

**Authors:** Pranav S. Ramesh, Li-Fang Chu

**Affiliations:** ^1^ Faculty of Veterinary Medicine, University of Calgary, Calgary, AB, Canada; ^2^ Reproductive Biology and Regenerative Medicine Research Group, University of Calgary, Calgary, AB, Canada; ^3^ Alberta Children’s Hospital Research Institute, Calgary, AB, Canada

**Keywords:** Notch signailing pathway, segmentation clock, gene oscillation, presomitic mesoderm (PSM), somitogenesis, somite

## Abstract

Somitogenesis is a hallmark feature of all vertebrates and some invertebrate species that involves the periodic formation of block-like structures called somites. Somites are transient embryonic segments that eventually establish the entire vertebral column. A highly conserved molecular oscillator called the segmentation clock underlies this periodic event and the pace of this clock regulates the pace of somite formation. Although conserved signaling pathways govern the clock in most vertebrates, the mechanisms underlying the species-specific divergence in various clock characteristics remain elusive. For example, the segmentation clock in classical model species such as zebrafish, chick, and mouse embryos tick with a periodicity of ∼30, ∼90, and ∼120 min respectively. This enables them to form the species-specific number of vertebrae during their overall timespan of somitogenesis. Here, we perform a systematic review of the species-specific features of the segmentation clock with a keen focus on mouse embryos. We perform this review using three different perspectives: Notch-responsive clock genes, ligand-receptor dynamics, and synchronization between neighboring oscillators. We further review reports that use non-classical model organisms and *in vitro* model systems that complement our current understanding of the segmentation clock. Our review highlights the importance of comparative developmental biology to further our understanding of this essential developmental process.

## Introduction

### Overview of the Notch signaling pathway

**FIGURE 1:**
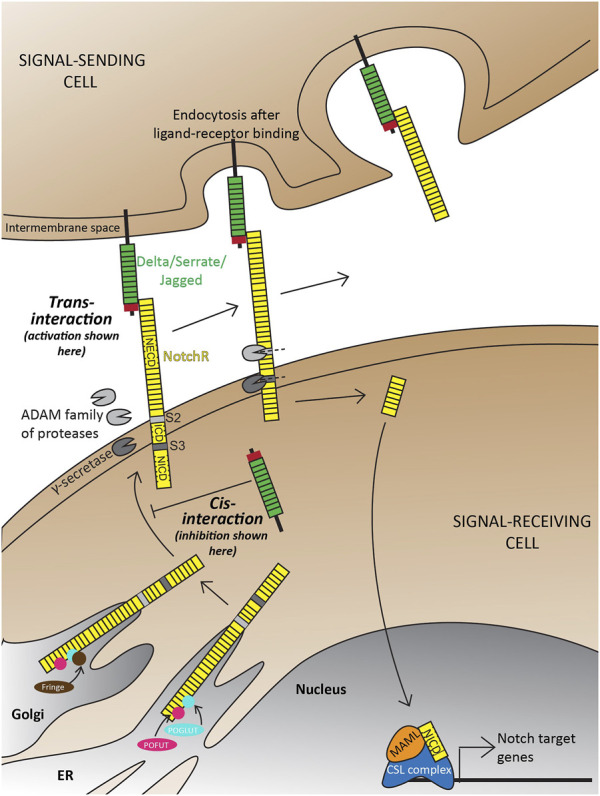
Overview of the Delta-Notch signaling pathway depicting *trans*-activation and *cis*-inhibition. Trans-activation is initiated when the DSL domain of the Delta/Serrate/Jagged ligands binds to the extracellular domain of Notch receptors. This initiates two crucial cleavages-the S2 cleavage by the ADAM protease; and subsequently an S3 cleavage by gamma-secretase. The two cleavages result in the production of free NICD that translocates into the nucleus to initiate transcription of target genes. Notch receptors, with are also a target of Notch signaling are subject to a wide range of post-translational modifications in the trans ER-Golgi space by Protein O-Fucosyltransferase (POFUT), Protein O-glucosyltransferase (POGLUT), Lunitic fringe (*Lfng*), *etc.*, which subsequently affect the type of ligand-receptor interactions and downstream signaling. Illustrations adapted and modified from (Zhou et al., 2022).

The canonical Notch signaling pathway is characterized by the activation of receptors on signal-receiving cells by ligands on signal-sending cells (Figure 1; Reviewed extensively in Kopan and Ilagan, 2009; Siebel and Lendahl, 2017; Zhou et al., 2022). In principle, the interacting extracellular domain of Notch ligands consists of a di-sulfide-rich DSL (Delta in mammals; Serrate in *Drosophila*; Lag-2 in *C. elegans*) domain and multiple tandem Epidermal Growth Factor (EGF) repeats. Notch receptors consist of a Notch Extracellular Domain (NECD), a Transmembrane Domain (TM), and a Notch Intracellular Domain (NICD) that are processed and fused in the endoplasmic reticulum and Golgi. Upon ligand-receptor binding, endocytosis is initiated in the signal-sending cell that exposes a characteristic S2 cleavage site in the NECD to the ADAM (A Disintegrin and Metalloprotease) family of proteases in the extra-cellular matrix (ECM). Subsequently, an S3 cleavage in the signal-receiving cell by the γ-secretase complex composed of Presenilin (Psen) 1/2, Nicastrin, APH1, and Presenilin enhancer (Psenen) releases the NICD to translocate into the nucleus. Here, NICD binds to a DNA-binding CSL complex (CBF1/RBPj in mammals, Suppressor of Hairless in *Drosophila*, Lag-1 in *C. elegans*) and a transcriptional coactivator mastermind-like (Maml 1–3) to initiate transcription of Notch target genes. In the absence of NICD, the CSL complex acts as a transcriptional repressor. Alternatively, a Notch ligand can bind the receptor intracellularly and prevent its trafficking to the cell surface thereby inhibiting the activation of this pathway (Figure 1). Both the activation and inhibition of this pathway are necessary for context-dependent biological processes (Reviewed extensively in Henrique and Schweisguth, 2019).

Although the fundamental components of this pathway remain conserved across different species, the numbers and function of Notch receptors and ligands vary greatly across model organisms due to the differential degrees of genome duplications and loss across species (Table 1).

**TABLE 1:** The number and types of Delta ligands and Notch receptors vary across species.

Notch ligands		
Species	Gene	References
Mouse/Human	*Dll1, Dll3, Dll4, Jag1, Jag2*	Bettenhausen et al. (1995), Lindsell et al. (1995), Shawber et al. (1996), Dunwoodie et al. (1997), Yoneya et al. (2001)
Chick	*delta1, serrate1, serrate 2*	Henrique et al. (1995), Myat et al. (1996), Vargesson et al. (1998), Caprioli et al. (2002)
Zebrafish	*deltaA, deltaB, deltaC, deltaD, dll4, jag1a, jag1b, jag2a, jag2b*	Haddon et al. (1998); Smithers et al. (2000), Lorent et al. (2004), Leslie et al. (2007)

Notch receptors		
Species	Gene	
Mouse/Human	*Notch1, Notch2, Notch3, Notch4; NOTCH2NLA/B/C* (Human)	del Amo et al. (1993), Fiddes et al. (2018); Higuchi et al. (1995), Lardelli et al. (1994), Robbins et al. (1992), Suzuki et al. (2018)
Chick	*notch1, notch2, notch3, notch4*	Vargesson et al. (1998); Williams et al. (2009)
Zebrafish	*notch1a, notch1b, notch2/notch6, notch3/notch5*	Bierkamp and Campos-Ortega (1993), Westin and Lardelli (1997)

### Modes of regulation of the Notch pathway- a brief introduction

There are several layers of Notch signaling regulation, which have been extensively reviewed in the past (Kopan and Ilagan, 2009; Henrique and Schweisguth, 2019). Here, we will briefly introduce two, which will be complemented later in the review with a more focused view in the context of species-specificity and early development.

The first layer of regulation is at the level of Notch ligand-receptor interactions, which can happen in *trans* (ligand and receptors present on different cells; Figure 1), or *cis* (ligand and receptor on the same cell; Figure 1) (Jacobsen et al., 1998; Artavanis-Tsakonas et al., 1999; Hicks et al., 2000; Sprinzak et al., 2010; Nandagopal et al., 2019), depending on the Notch ligand-receptor pair interacting. For example, *in vitro* culture studies using immortalized cell lines have shown that Dll1 and Dll4 can act as *trans*-activating or *cis*-inhibiting ligands (Andrawes et al., 2013; Preuße et al., 2015), whereas Dll3 has been shown to act as a *cis*-inhibiting ligand by physically binding to Notch1 and blocking Notch signaling both *in vitro* in HEK293 cells and *in vivo* during both *X. laevis* and mouse neurogenesis (Ladi et al., 2005). Dll4 has been shown to have a strong *cis*-inhibitory effect on Notch1 receptors which can partially override Dll1-mediated trans-activation *in vitro* using HeLa cells and Chinese Hamster Ovary cells (Preuße et al., 2015). Another study, which also used immortalized HeLa cells *in vitro*, showed that Dll3 can act in *cis*, and potentiate the *trans*-activating capabilities of Dll1 (Bochter et al., 2022). While *trans* and *cis*-regulation have been mechanistically demonstrated, it is still challenging to generalize the activating/inhibiting effects of different ligands observed in the above studies to particular tissue- or species-specific contexts. For example, mutant mice carrying knock-in *Dll4* in the endogenous *Dll1* locus demonstrated that Dll1 and Dll4 are completely interchangeable during early retinal development, partially interchangeable during myogenesis, and not interchangeable during somitogenesis (Preuße et al., 2015).

The second layer of Notch signaling control arises through post-translational modifications of Notch receptors and ligands (Figure 1). Some of these include o-glucosylation, o-fucosylation, or N-Acetylglucosamination of consensus residues in the EGF repeats that allow a cell to distinguish between different ligand-receptor interactions (Kakuda and Haltiwanger, 2017). For example, o-glucosylation and o-fucosylation of the Notch receptors are carried out by Protein O-Glucosyl Transferase (POGLUT) and Protein O-Fucosyl Transferase (POFUT) respectively in the trans-Golgi network (Shi and Stanley, 2003; Acar et al., 2008). These modifications act as a base for N-Acetylglucosamination imparted by Notch signaling targets called the *Fringe* proteins which modulate the receptor’s ability to respond to ligands like Dll1 or Jag1 (Kakuda and Haltiwanger, 2017; Bochter et al., 2022). Seemingly, both Dll1 and Dll3 also have these conserved residues, making them amenable to these modifications (Bochter et al., 2022). Expression of one of the *Fringe* proteins- Lunatic Fringe *(Lfng)* in a signal-sending cell (containing the trans-activating ligand Dll1) has been shown to attenuate Notch-activation *in vitro* using HEK293T and NIH3T3 cells (Bochter et al., 2022). *In vitro* studies using immortalized cell lines have also shown that Lfng can potentiate Notch activation by Dll1, but inhibit activation by Jag1 (Brückner et al., 2000; Yang et al., 2005; Kakuda and Haltiwanger, 2017). This is intriguingly in contrast to *in vivo* data during somitogenesis between E8.75-E11.5, where *lfng* (the only *Fringe* gene expressed during mouse somitogenesis (Johnston et al., 1997)) can post-translationally inhibit NICD formation from the Notch1 receptor, thereby generating a cyclic NICD profile (Morimoto et al., 2005; Shifley et al., 2008). These results suggest that post-translational modifications imparted by Lfng on Notch receptors and ligands can be lineage-specific, and their consequences can be highly context-specific, for example, if Lfng is present in the signal-sending/receiving cell.

Notch signaling, and these different modes of regulation play crucial roles during animal development, but more importantly, are crucial for proper somitogenesis (Reviewed in Hubaud and Pourquié, 2014; Oates et al., 2012). In the following sections, we will review this developmental process, its history, and the complexity it involves at both the tissue and cellular levels.

### Somitogenesis and the segmentation clock

The process of somitogenesis is an early developmental event occurring between the human embryo Carnegie stage 9 to stage 13 or within the first month post-fertilization (Orts-Llorca, 1981; O’Rahilly and Müller, 2001; O’Rahilly and Müller, 2010). However, knowledge about this process in human embryos has been largely lacking and most of what we have learned to date has been through classical model organisms like chick, mouse, and zebrafish. Most recently however, single-cell RNA sequencing data from early human gastrulating embryos has helped us infer critical information about human somite formation (Xiang et al., 2019; Tyser et al., 2021; Zeng et al., 2023).

Somitogenesis involves the sequential formation of transient, block-like structures called somites that eventually give rise to the bones, skeletal muscles, and dermis associated with the vertebrae. Somites differentiate from the presomitic mesoderm (PSM) tissue that spans along the anterior-posterior axis of a developing embryo. In this tissue, somites bud off of the anterior PSM, while new mesoderm cells are being generated by the mesoderm progenitors at the posterior end (or the tail bud) (Chalamalasetty et al., 2014; Row et al., 2016). The rates of these two processes at opposing ends of the PSM regulate the shrinkage of the tissue over time. Once the tissue is exhausted, somitogenesis is terminated, resulting in a species-specific duration of somitogenesis and somite numbers (Yoshikawa et al., 1997; Yoon and Wold, 2000; Ciruna and Rossant, 2001; Fior et al., 2012).

Pioneering work regarding the mechanisms underlying somitogenesis used chick as a model system with crucial speculations made regarding the origin of somites. One of the many views postulated the existence of a somitic ‘pre-pattern’ in the primitive streak that gets translated into the somite series produced by the PSM (Pasteels, 1937; Waddington, 1952). These views were backed by fate-mapping techniques in both chick and mouse that suggested the presence of stem-like cells in the primitive streak whereby their position along the A-P axis defined the future organization of the somitic tissue (Selleck and Stern, 1991; Psychoyos and Stern, 1996; Wilson and Beddington, 1996; Eloy-Trinquet and Nicolas, 2002).

**FIGURE 2:**
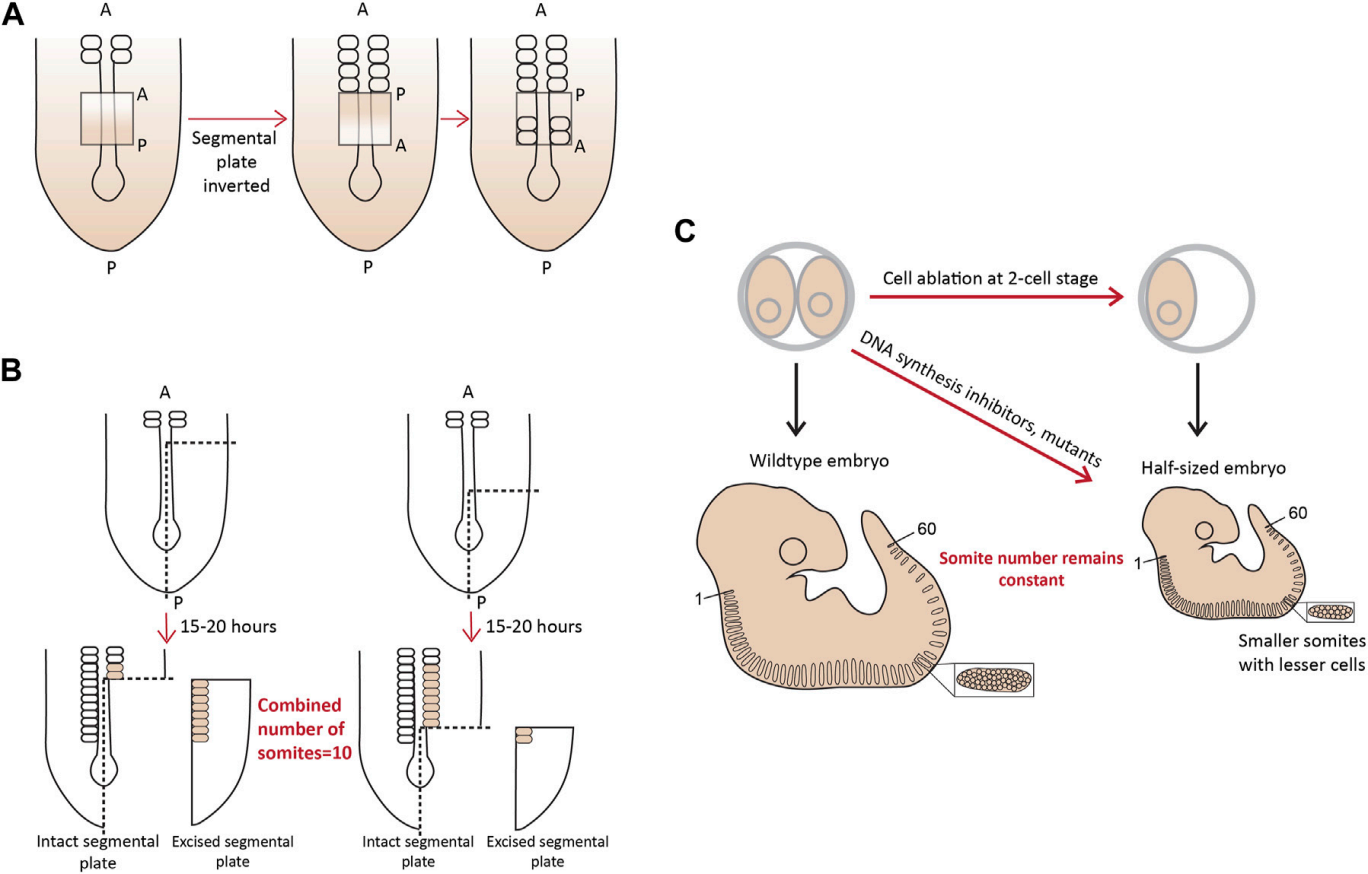
A molecular prepattern underlies the specification of PSM cells from the tail bud **(A)**. Inversion of the unsegmented chick PSM results in this tissue forming somites in the opposite direction **(B)**. An explant of chick segmental plate will form 10 somites irrespective of the site/timing of cutting **(C)**. A smaller mouse embryo will form shorter somites, but maintain the mouse-specific somite number. Illustrations adapted and modified from (Pourquié, 2004).

More evidence for the existence of a pre-pattern came when a part of the chick PSM, if surgically inverted even as early as Hamburger-Hamilton (HH) stage 2 (15–20 somite stage), formed somites in an opposite posterior to anterior fashion (Figure 2A; Christ et al., 1974; Palmeirim et al., 1998; Menkes and Sandor, 1977). Additionally, when approximately one-fourth of a chick embryo at the gastrula stage was excised, or mutant mouse embryos with a shorter axis were analyzed around E10.5, the number of somites formed remained the same, with each new somite also being shorter containing fewer cells (Waddington and Deuchar, 1953; Flint et al., 1978). Landmark experiments performed using chick and snapping turtle embryos, where variable lengths of the segmental plate were excised and cultured *in vitro* showed that the number of somites formed from the intact segmental plate in the embryo and the explant, combined, always remained constant-about 10–12 somites (Figure 2B; Packard, 1978; Packard, 1980). Important work from Tam and others in the 1980s showed similar compensatory associations in mouse embryos. When one of the cells at the 2-cell blastula stage was surgically removed, or embryos were treated with DNA replication inhibitors resulting in smaller, half-sized embryos, but having the conserved number of somites (Figure 2C; Tam, 1981). Similar results have been reported in small-sized zebrafish embryos, which show a smaller somite size such that the somite number remains unchanged (Ishimatsu et al., 2018). Another recent study has also re-enforced the view of a pre-pattern by performing grafts of the primitive streak (PS) from older (HH8) to younger (HH4) chick embryos (Busby et al., 2023). While GFP^+^ grafts from an HH4 PS to an HH4 PS appeared in more anterior (first formed) somites, grafts from an HH8 PS transplanted into HH4 only populated the posterior (later formed) somites. This revealed that the progenitor addition to the PSM is regulated by an intrinsic timer or a definitive prepattern (Busby et al., 2023).

**FIGURE 3:**
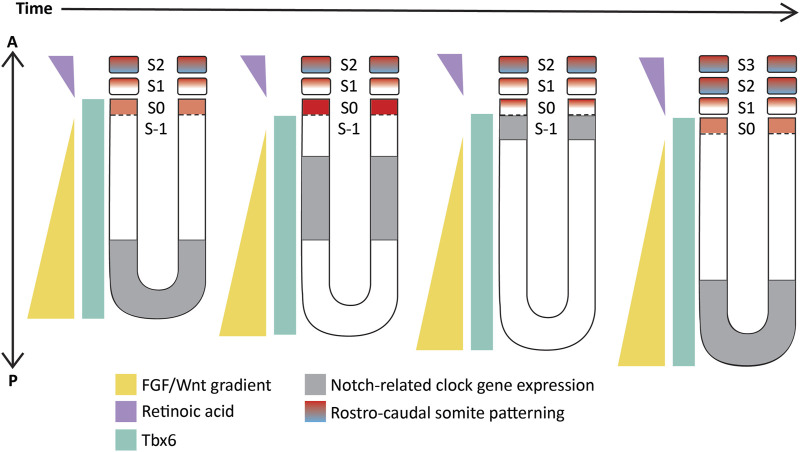
Schematic representation of the ‘Clock-and-wavefront’ model that shows the existence of a clock travelling from the posterior to the anterior PSM (in grey) coupled with a regressing wavefront (in yellow) which interact precisely with another T-box TF, Tbx6 (in cyan) to specify anterior PSM cells to differentiate into a somite. S-1: unsegmented PSM that will form the next somite; S0- The somite that is being specified; S1- The most recently formed somite; S2- The earliest formed somite that has completed rostro-caudal patterning (blue-red gradient). Illustrations adapted and modified from (Pourquié, 2011).

**FIGURE 4:**
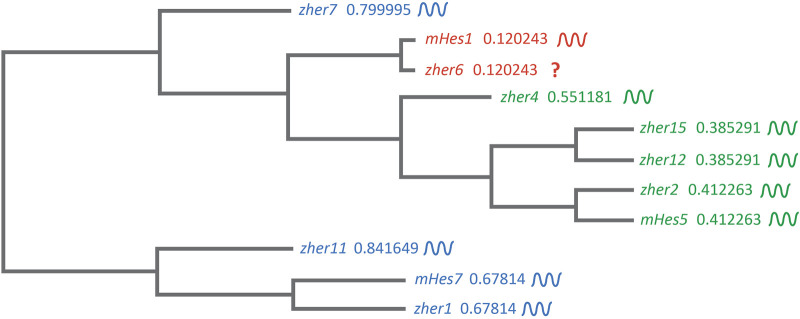
Clustal-based phylogenetic tree of mice (m) and zebrafish (z) *Hes1/5/7* orthologs. Amino acid sequences of *mHes7* (NP_149030.2)*, mHes5* (NP_034549.1)*, mHes1* (P35428) *zher1* (Q90463)*, zher7* (Q9I9K1)*, zher11* (Q6W4T8)*, zher2* (Q90464)*, zher4* (Q90466)*, zher12* (Q6TA36)*, zher15* (Q7T3J0), and *zher6* (Q6PBX3) were first aligned in the FASTA format using MUltiple Sequence Comparison by Log- Expectation (MUSCLE). The FASTA alignment file was input into the EMBL-EBI Simple Phylogeny tool. The tree was generated in a Clustal format with distance corrections and a UPGMA (Unweighted Pair Group Method with Arithmetic Mean) clustering method. The phylogenetic tree depicts the separation of the *Hes1*, *Hes5,* and *Hes7* clusters. The symbol beside each gene shows their expression dynamics in the PSM, which is unclear specifically for *her6* as it is expressed only during the 3-5 somite stage during fish segmentation.

These studies showed and strongly corroborated the existence of a molecular pre-pattern that regulates the metameric organization program of somites. To explain its molecular basis, Cooke and Zeeman in 1976 theoretically postulated the presence of the classical ‘Clock-and-Wavefront’ model (Cooke and Zeeman, 1976; Reviewed in Resende et al., 2014). It predicted that the formation of repeated patterns with high precision is possible if the developing tissue consists of a “clock” made up of individual phase-locked cellular oscillators that interact with a slowly regressing front generated by a signaling gradient to change the cell state (Figure 3). It was not until 1997 when the first experimental evidence for the clock surfaced in the chick embryo, and was termed the ‘Segmentation clock’ (Palmeirim et al., 1997). Notch targets *c-hairy1/2* which belong to the Hairy/enhancer of split (*Hes/her/Hey*) group of basic-helix-loop-helix (bHLH) transcription factors (TFs) were shown to oscillate in the PSM. These oscillations occurred in a spatiotemporally coordinated manner resulting in “traveling” waves sweeping in a posterior-to-anterior direction once for every somite with a periodicity of 90 min (Palmeirim et al., 1997; Pourquié and Tam, 2001), matching the time taken to form one somite in chick, a process that begins at around HH stage 7. Oscillatory *Hes/her/Hey* genes are also present in mice and zebrafish that make up the clock during somitogenesis (Table 2) (Reviewed in detail in Kageyama et al., 2007; Maroto et al., 2008). These genes have orthologous and paralogous relationships to one another given the large number of gene duplications in zebrafish (Figure 4; Taylor et al., 2001), and are critical to the segmentation clock to varying degrees as we will discuss later below. Readers interested in a detailed analysis of these orthologous relationships and their evolution can refer to (Zhou et al., 2012; Kuretani et al., 2021).

**TABLE 2:** PSM-specific Notch signaling-related *Hes/her/Hey* genes in mouse, zebrafish, and chick.

Species	PSM-expressed *hes/her/hey* genes			PSM expression profile		References	
Mouse	*Hes1*			Cyclic		Jouve et al. (2000)	
	*Hes5*			Cyclic		Del Barco Barrantes et al. (1999)	
	*Hes7*			Cyclic		Bessho et al. (2001a)	
	*Hey1*			Cyclic or non-cyclic?		Leimeister et al. (2000), Krol et al. (2011)	
	*Hey2*			Cyclic		Leimeister et al. (2000)	
Zebrafish	*her1*	*mHes7* orthologs		Cyclic		Holley et al. (2000); Holley et al. (2002)	
	*her7*			Cyclic		Oates and Ho (2002), Gajewski et al. (2003)	
	*her11*			Cyclic		Sieger et al. (2004)	
	*her2*	*mHes5* orthologs		Cyclic		Krol et al. (2011)	
	*her4*			Cyclic		Krol et al. (2011)	
	*her12*			Cyclic		Shankaran et al. (2007)	
	*her15*			Cyclic		Shankaran et al. (2007)	
	*her6*	*mHes1* ortholog		Expressed in the PSM between 3–5 somite stages		Pasini et al. (2004)	
	*her13.2*	*mHes6* ortholog		Posterior-Anterior gradient		Kawamura et al. (2005)	
	*hey1*	*mHey1* ortholog		Cyclic only in rostral PSM		Winkler et al. (2003)	
Chick	*c-hairy1*			Cyclic		Palmeirim et al. (1997)	
	*c-hes1*			Cyclic		Krol et al. (2011)	
	*c-hey1*			Lowly expressed in caudal PSM		Leimeister et al. (2000)	
	*c-hairy2*			Cyclic		Jouve et al. (2000)	
	*c-hey2*			Cyclic		Leimeister et al. (2000)	
	*c-hes5*			Cyclic		Krol et al. (2011)	

Despite the elegance of the clock-and-wavefront model in explaining pattern formation during somitogenesis, certain properties of the segmentation clock make this process more complex than this proposed model.

## Complexities beyond the clock-and-wavefront model

### Determination front: gradient or cyclic?

**FIGURE 5:**
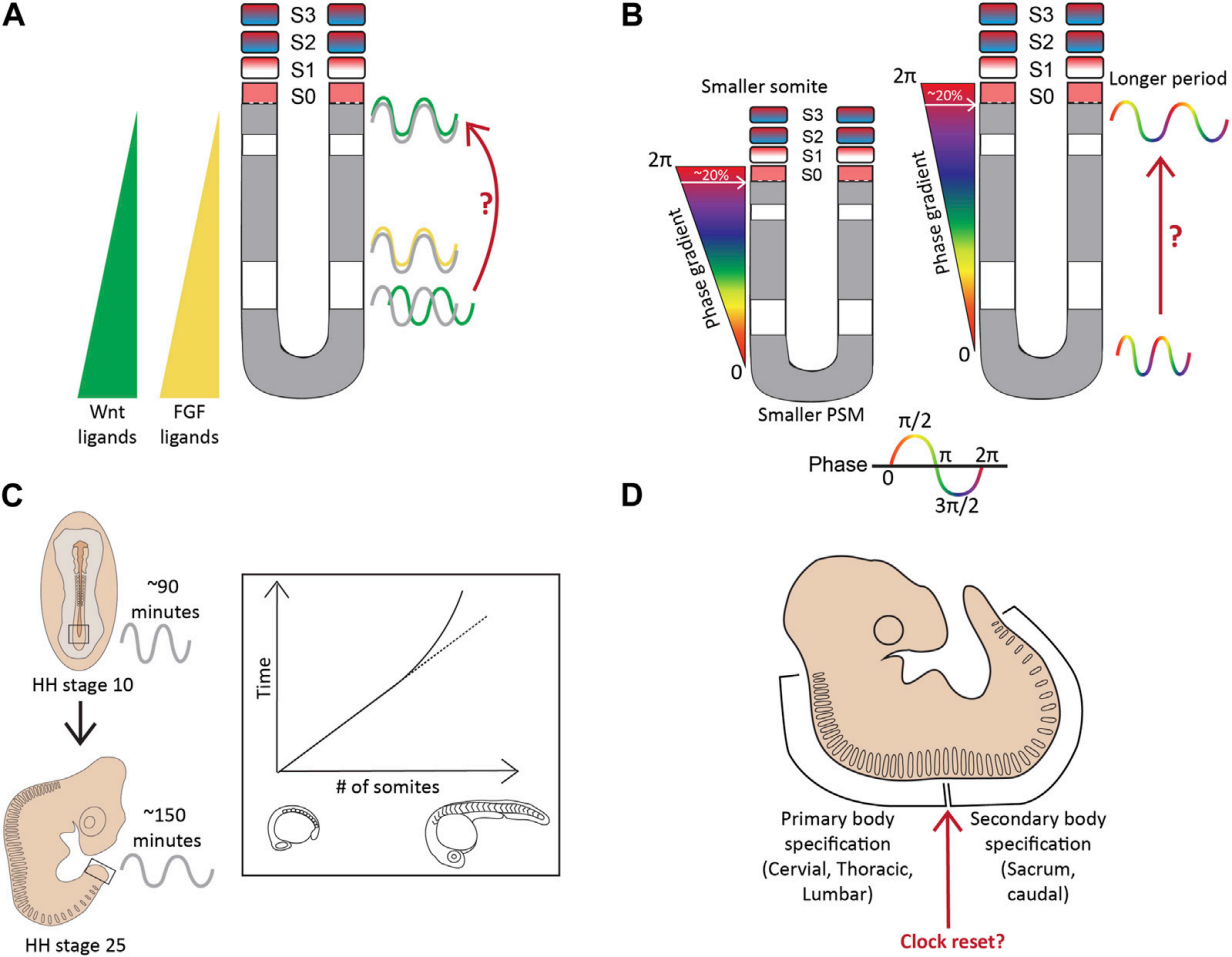
Complexities beyond the clock and wavefront **(A)**. The regressing Wnt/FGF-based gradients from the posterior to anterior PSM specify the determination front, however, targets of these signaling pathways (green-Wnt; yellow-FGF) oscillate in the PSM, with changing phase relationships to Notch signaling targets (grey) **(B)**. The period and the phase of cyclic gene oscillations change along the length of the PSM **(C)**. The period of the segmentation clock changes temporally as somitogenesis proceeds **(D)**. The anterior and posterior body segmentation are plausibly regulated by separate mechanisms. Illustrations adapted and modified from (Schröter et al., 2008; Lauschke et al., 2012).

Classically, the clock-and-wavefront model suggests the presence of regressing FGF/WNT gradients in the PSM that act as the ‘determination front’ determine the somite borders and the pace of somitogenesis (Figure 3; Sawada et al., 2001; Naiche et al., 2011; Akiyama et al., 2014). However, many studies have shown oscillating FGF and Wnt signaling targets in the caudal part of the mouse E8.5-10.5 PSM such that Notch and FGF targets oscillate in-phase, and out-of-phase of Wnt targets (Figure 5A; Dequéant et al., 2006; Hayashi et al., 2009). Furthermore, Notch and Wnt targets oscillate in-phase in the rostral E10.5 PSM when grown *ex vivo* as 2D cultures and disrupting this spatial relationship between the two pathways can disrupt somitogenesis (Sonnen et al., 2018). In addition, Hes7 has been shown to regulate the expression of FGF and Wnt target genes like *Dusp6, Sprouty2, Axin2*, and *Snai1* during somitogenesis. For example, studies showed that Hes7 represses the expression of FGF targets, *Sprouty2* and *Dusp4*, in the E10.5 mouse PSM (Niwa et al., 2007; Hayashi et al., 2009). One study showed that disrupting *Hes7* cycling by changing gene length disrupted *Axin2* and *Dusp6* cyclic expression in the E10.5 PSM (Takashima et al., 2011). On the contrary, another study showed that *Hes7* ^*−/−*^ embryos still display dynamic Wnt (*Axin2*) and FGF (*Sprouty2*) target gene oscillations (Ferjentsik et al., 2009). This raises the question as to whether or not Wnt and FGF signaling oscillate or slowly regress as a gradient, or both (Figure 5A). Therefore, other models like the ‘two-phase model’ or the ‘phase-shift model’ have also been suggested that incorporate an interacting Notch, FGF/Erk, and Wnt oscillator (Reviewed in detail in Hubaud and Pourquié, 2014). Whether these expression patterns co-exist during the segmentation clock or are mutually exclusive remains to be resolved (Aulehla et al., 2003). It is possible to speculate that the wavefront is oscillatory under the control of *Hes* genes in the middle and anterior PSM but acts as a gradient in the more posterior tissue.

**FIGURE 6:**
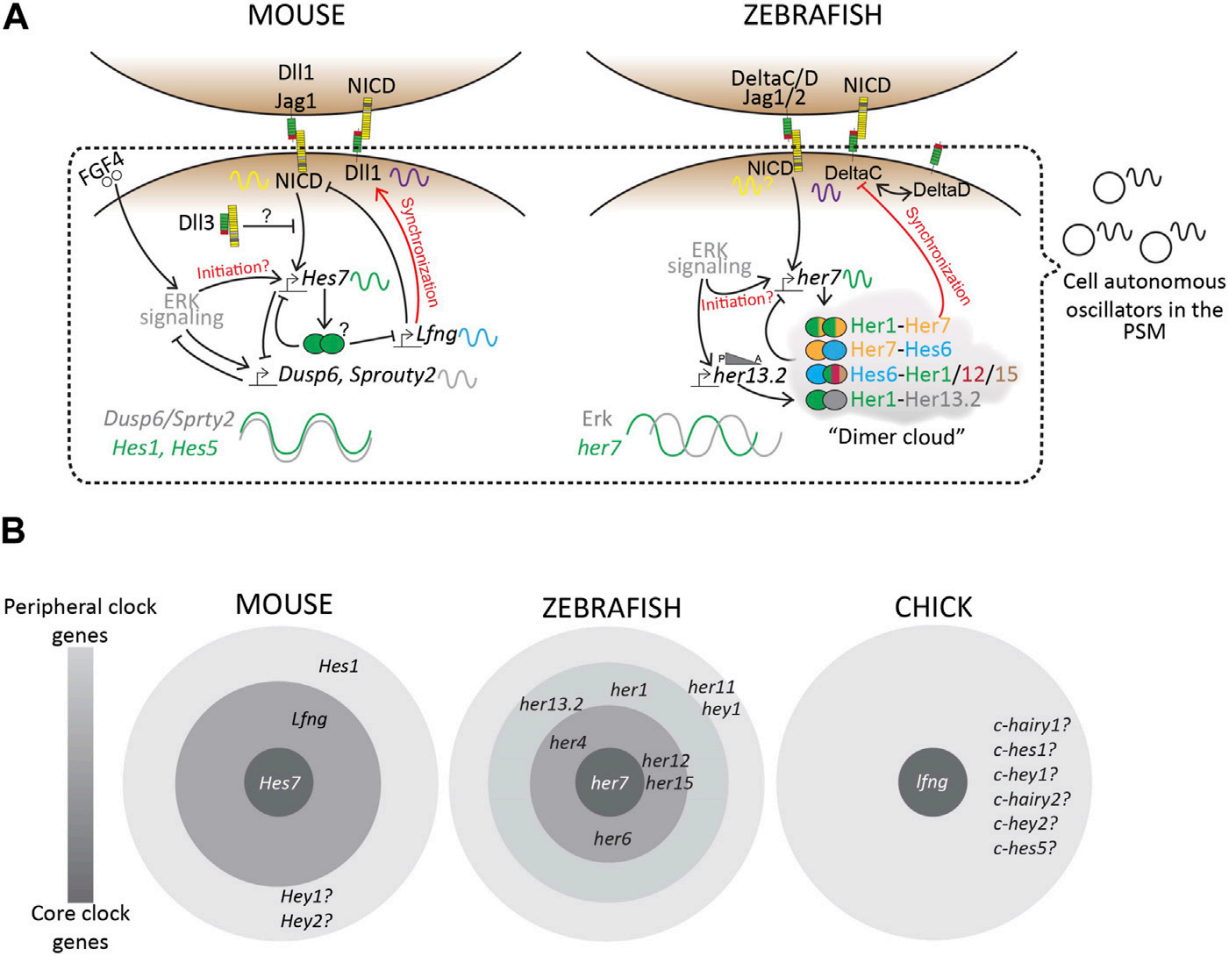
Transcriptional landscape of the segmentation clock. **(A)** A simplified schematic of the complex regulatory landscape underlying the mouse and zebrafish segmentation clocks. **(B)** “Core” and “Peripheral” bHLH genes and *Lfng*, amongst many others, occupy this regulatory landscape. Based on mutant phenotypes, these clock genes regulate the segmentation clock to varying degrees. Zebrafish consists of many more *her/hey* genes as a part of its segmentation clock, as compared to mouse or chick, plausibly making the zebrafish clock more robust to genetic or environmental perturbations compared to other species. Finally, we know very little about similar core vs. peripheral clock genes in other species including higher vertebrates like humans, resulting in a knowledge gap that needs to be studied to fully understand this biological clock.

More importantly, a recent study on the zebrafish posterior PSM showed that, unlike mice, Notch-target *her7* and Erk signaling oscillate out of phase (Figure 6A; Simsek et al., 2023). This species-specific difference adds an additional layer of complexity to uncovering the mechanisms underlying these phase relationships between the different signaling pathways.

Another deviation from the classic clock-and-wavefront model, that emerged in the decade following its discovery was that the clock is not constant, but rather highly dynamic at both the spatial and temporal axes.

### Changing periodicity of the segmentation clock spatially along the A-P axis

Firstly, the period of the segmentation clock varies along the A-P axis of the PSM, slowing down in the anterior PSM, thereby generating waves of cyclic gene expression (Figure 5B). This phenomenon is apparent in the mouse and zebrafish PSM (Soroldoni et al., 2014; Shih et al., 2015; Liao et al., 2016; Tsiairis and Aulehla, 2016; Liao and Oates, 2017). In a 10–12 somite-stage zebrafish embryo, the *her1* clock in the posterior PSM oscillates with a 1-somite periodicity (30 min), whereas with a slower 2-somite periodicity (∼60 min) in the anterior PSM (Shankaran et al., 2007; Shih et al., 2015). In mice, the period increases from ∼130 min in the posterior PSM to ∼170 min in the anterior PSM as assayed by oscillations of another clock gene *Lfng* in E10.5 2D PSM explants (Tsiairis and Aulehla, 2016). Alongside a period gradient, there also exists a ‘phase gradient’ such that somite formation in the anterior PSM always coincides with a 2π phase-shift of the clock compared to the posterior PSM (Lauschke et al., 2012). Essentially, this means that the cells in the anterior PSM are one cycle apart from the cells present posteriorly, reiterating the passing of one cycle for every somite formed. Additionally, a newly formed somite always spans ∼20% of this phase-gradient irrespective of PSM length (Figure 5B; Lauschke et al., 2012). In other words, a smaller PSM would form a “steeper” phase gradient to maintain this 2π phase shift, and as a result, 20% of this gradient results in smaller segments (Figure 5B). This presents a plausible mechanism underlying smaller-sized embryos forming the same number of (and smaller) somites.

One potential consequence of such a period/phase gradient has been hypothesized based on the existence of a “secondary” non-changing oscillator along the length of the PSM (Goodwin and Cohen, 1969; Lauschke et al., 2012). The phase of the segmentation clock changes as cells move along the PSM, and its relative phase difference with this secondary oscillator can help give cells the necessary spatial (alongside temporal) information to differentiate into a somite. One potential candidate for this secondary oscillator in mice is Wnt signaling. As mentioned in the previous sub-section, disrupting the phase relationship between Notch and Wnt oscillations results in disorganized somitogenesis. In zebrafish, one candidate is *her12* whose phase relationship with *her1* also changes along the PSM (Shankaran et al., 2007). However, whether or not these candidate oscillators also display a period/phase gradient along the PSM needs to be systematically characterized.

But what is the cause underlying this gradient? Chemical-based reduction in the levels of Wnt signaling in both mouse and chick PSM slows down the clock, forming one fewer somite compared to the untreated controls (Gibb et al., 2009). The Wnt3a ligand exists as a gradient in the PSM (Aulehla et al., 2003; 2008), and as a result, reducing levels of Wnt signaling along the length of the PSM could be one potential reason for the period gradient.

Perhaps, understanding the molecular and genetic mechanisms underlying the period gradient within a particular species could help us better understand how and why the clock time differs across different species.

### Temporal alteration in the segmentation clock period

The period of the clock also changes as segmentation proceeds, remaining rather constant for most of the duration of somite formation, but drastically slowing down by ∼1.5-fold for the last few somites in both chick and zebrafish (Figure 5C; Schröter et al., 2008; Gibb et al., 2009; Tenin et al., 2010). This layer of complexity has been less characterized during mouse segmentation. The time taken for somite formation in chick embryos between HH22-HH24 decelerates from 90 min to ∼150 min, and this slowing correlates with when Wnt3a levels change from a gradient in the PSM to being confined to the tip of the tail bud to finally disappearing (Gibb et al., 2009; Tenin et al., 2010). During this time, most if not all Notch activity is also lost in the PSM and the clock terminates before all somites are formed at HH25, suggesting a clock-independent regulation of somite formation towards the later stages of development (Tenin et al., 2010). Interestingly, something similar is observed in mouse embryos at E12.5 when all Wnt activity is restricted to the caudal tip and is completely lost at E13.5 before somitogenesis ceases at E14.5, suggesting a similar temporal alteration of the clock during mouse somitogenesis (Gibb et al., 2009). These results also suggest the intriguing possibility that the slower mouse clock (compared to chick) is due to the mouse PSM intrinsically expressing lower levels of Wnt ligands/effectors compared to the chick PSM.

### Separate mechanisms regulating the clock along the A-P axis

Another layer of complexity in the process of somitogenesis arises from the speculations that the mechanisms regulating this process differ during anterior and posterior body specification (Figure 5D). The switch between primary and secondary body segmentation in mice occurs at the lumbosacral junction (Gossler and Tam, 2002).

Both *Dll3* ^*−/−*^ and *Lfng* ^*−/−*^ mice have highly disorganized thoracic and lumbar vertebrae, however, *Lfng* ^*−/−*^ mice can produce several normal sacral vertebrae before forming a disorganized sacrum and a short tail (Stauber et al., 2009; Williams et al., 2014; 2016; Bochter et al., 2022). *Dll3* ^*−/−*^ *; Lfng* ^*−/−*^ mice produced no normal sacral vertebrae, suggesting that while *Lfng* and *Dll3* may work together during anterior body specification, they exhibit independent roles posteriorly. Furthermore, the deletion of an enhancer Fringe clock element 1 (FCE1) upstream of *Lfng* drastically reduced *Lfng* expression in the caudal E10.5 PSM while maintaining its anterior expression. *Lfng* ^*ΔFCE1/ΔFCE1*^ embryos phenocopied *Lfng* ^*−/−*^ embryos during anterior segmentation including heavily disorganized and fused somites and missing ribs, but formed normal tail vertebrae (Shifley et al., 2008). qRT-PCR showed, unlike *Lfng* ^*−/−*^, *Lfng* ^*ΔFCE1/ΔFCE1*^ embryos still showed very low cyclic *Lfng* activity (Shifley et al., 2008; Williams et al., 2014). Similarly, while *Dll1* ^*−/−*^ mouse embryos fail to form somites throughout the body axis (De Angelis et al., 1997), embryos homozygous for a dominant negative allele of *Dll1* can segment, but show fused/missing and split somites only during cervical and thoracic segmentation (Cordes et al., 2004). This suggests that while robust, high-level cyclic *Lfng* or Notch ligand function is crucial for primary body segmentation, this necessity is no longer present posteriorly, indicating a switch in mechanisms underlying the segmentation clock. Finally, FGF4 mouse mutants also show disorganized somite specification only during the 5-6 somite stage (E8.5) but show normal somitogenesis during the 24–26 somite stage (E9.5), corroborating this complexity (Anderson et al., 2020).

As we will also discuss later below, *her1* ^*−/−*^ zebrafish embryos, or *her1*-morpholino treated embryos show minor segmentation defects with diffuse somite borders only for the first 3-5 somites, while *her7* knockdown resulted in severe somite border defects after a 5-somite delay (Henry et al., 2002). Furthermore, *her1* ^*−/−*^ *;her7* ^*−/−*^ embryos do not form somites throughout the embryonic axis (Henry et al., 2002). Inserting *her1* and *her7* transgenes in single chromosomes (different from the endogenous locations) in these double-knockout embryos resulted in partially rescued posterior, but not anterior segmentation (Keseroglu et al., 2023). This again suggests separate mechanisms regulating somitogenesis along the embryonic axis. These complexities arise as a result of intercommunication between the Notch, Wnt, and FGF signaling pathways.

In the following sections, we review the species-specific Notch signaling dynamics of the segmentation clock with a focus on bHLH-TFs, ligand-receptor functions, and how these dynamics are propagated by Notch-mediated cell synchronization. Readers interested in a detailed review specifically on the zebrafish segmentation clock can refer to (Venzin and Oates, 2020).

## bHLH-transcription factors as the core regulators of the segmentation clock

The Notch-responsive family of bHLH-TFs are transcriptional repressors that can bind to the E-box or N-box regions of certain promotors and suppress gene expression (Bessho et al., 2001b; Jones, 2004; Chen et al., 2005). Some of these transcription factors also bind to their own promoters to generate molecular oscillations via a negative feedback loop in the PSM (Holley et al., 2002; Bessho et al., 2003; Jones, 2004; Giudicelli et al., 2007). Mutating these binding sites on target promoters prevents this binding *in vitro* (Chen et al., 2005). Murine orthologs of Notch-responsive bHLH TFs *c-hairy* (*Hes/Hey* genes) include *Hes1-7*, *Hey1-2*, and *HeyL,* of which, *Hes1, Hes5*, *Hes7,* and *Hey2* are expressed cyclically in the E8.5-E10.5 mouse PSM (Del Barco Barrantes et al., 1999; Jouve et al., 2000; Leimeister et al., 2000; Bessho et al., 2001b). *Hey1* transcripts can also be detected in the mouse E9.5 embryos by *in situ* hybridization, but are not oscillatory (Leimeister et al., 1999). However, microarray experiments have shown that *Hey1* indeed oscillates in the mouse E9.5 PSM (Krol et al., 2011). Similar to the first described expression patterns in chick embryos, mouse embryos also show autonomous traveling waves of cyclic gene expression sweeping along the PSM with a mouse-specific periodicity of 2 h (Aulehla et al., 2008). In fact, the *Hes1* gene has also been shown to cycle with a conserved 2-h periodicity in other cell types like neural progenitor cells *ex vivo*, fibroblasts, myoblasts, and embryonic stem cells *in vitro*, suggesting that a species-specific ultradian rhythm of these bHLH-transcription factors is a conserved, vital feature of vertebrate development (Hirata et al., 2002; William et al., 2007; Ochi et al., 2020). This has been reviewed in detail (Carraco et al., 2022).

Interestingly, a high divergence has been observed *in vivo* and *in silico* between chick, mouse, and zebrafish when comparing oscillating genes in the PSM (Krol et al., 2011). Specifically, while the Notch, Wnt, and FGF signaling pathway targets oscillate in all three species, the cyclic expression patterns of orthologs of only two genes- *Hes1* and *Hes5* are conserved (Krol et al., 2011). We speculate that comparisons across more species could reveal additional conserved oscillators. As we will discuss in the following sections, a comparative analysis of the various *Hes/her* genes reveal diverging core clock genes and their importance in regulating the segmentation clock. We will review, in-depth, the complex regulatory web casted by these transcription factors during somitogenesis with the anticipation of detangling this species-specific divergence.

### Loss of function phenotypes reveal core versus peripheral players of the mouse segmentation clock

While *Hes* genes are cyclically expressed in the mouse PSM, not all of them seem to be essential for maintaining the clock. For example, *Hes7* ^*−/−*^ E8.5 and E9.5 PSM lose all Notch-responsive cyclic activity, including that of other clock genes like *Lfng* and *Hes1*, and show misexpression of somite maturation markers resulting in disfigured fused somites (Table 3; Bessho et al., 2001a; Ferjentsik et al., 2009; Hirata et al., 2004). However, the expression of upstream components like the Delta ligands and Notch receptors is unaffected suggesting the hierarchy of Notch signaling (Bessho et al., 2001b). On the other hand, *Hes1* ^*−/−*^ mice still show cyclic *Lfng* activity in the E10.5 PSM and somite formation is unaffected (Jouve et al., 2000). *Lfng* ^*−/−*^ E9.5-E10.5 PSM also show cyclic *Hes7* and Notch activity, but form disfigured somites, a phenotype that is still less severe compared to *Hes7* ^*−/−*^ mice (Ferjentsik et al., 2009). Conceptually, this puts *Hes7* as a core clock gene, *Lfng* as a peripheral clock gene, and *Hes1* as a more peripheral gene (Figure 6B). In fact, the promoter of *Lfng* consists of N-box sequences to which Hes7 physically binds and represses expression (Chen et al., 2005). Interestingly, DNase-footprinting assays have shown that Hes1 only binds N-box sequences (and not E-boxes) to repress transcription (Sasai et al., 1992). Yet, Hes1 does not seem to affect *Lfng* transcription, indicating that these Notch signaling-related DNA-protein interactions are also highly lineage-specific. This kind of core vs. peripheral function may arise as a result of functional redundancy where the loss of *Hes1* is fully compensated by *Hes7*. However, what makes one *Hes* gene a core player over another remains elusive.

**TABLE 3:** Mice knockout phenotypes of PSM-specific *Hes/her/Hey* targets, Notch ligands, and receptors.

			PSM-specific expression										
Mouse mutants	Embryonic development	Somitogenesis	*Hes1*	*Hes5*	*Hes7*	*Hey1*	*Hey2*	*Dll1*	*Dll3*	*Jag1*	*Notch1*	*Notch2*	References
*Hes1^−/−^*	Embryos die neonatally by E12.5	Normal somite formation	NR (except PSM showing normal Lfng cycling)										Ishibashi et al. (1995), Jouve et al. (2000)
*Hes5^−/−^*	Normal	*Hes1^−/−^Hes5^−/−^* embryo show normal somitogenesis	NR										Ohtsuka et al. (1999)
*Hes7^−/−^*	Postnatal lethality	Somite-specific expression disrupted and fused, irregular somites resulting in shorter trunk	CL				CL	NA in PSM, CL in somite	UA with diffused borders		UA with diffused borders	UA with diffused borders	Bessho et al. (2001b), Ferjentsik et al. (2009)
*Hey1^−/−^*	Normal	Normal somite formation	NR										Fischer et al. (2004)
*Hey2^−/−^*	Postnatal lethality	*Hey1^−/−^Hey2^−/−^* show normal somite development	NR										Leimeister et al. (2000), Gessler et al. (2002)
*Dll1^−/−^*	Embryos die neonatally by E12	Severely disrupted borders, no somites/lineages formed	CL	CL	CL except caudally	CL	CL except caudally		NA at E9 UR at E10.5	DR			De Angelis et al. (1997); Del Barco Barrantes et al. (1999), Jouve et al. (2000), Leimeister et al. (2000), Niwa et al. (2007); Sewell et al. (2009), Preuße et al. (2015), Shimojo et al. (2016)
*Dll3^−/−^*	Postnatal lethality	Disorganized border but clear metameric units, somite lineages formed	CL except rostrally	CL	Normal: Cyclic/non-cyclic?	CL except rostrally		NA			∼1.3-fold DR; cleaved Notch1 (NICD) domain is expanded in the rostral PSM		Dunwoodie et al. (1997), Kusumi et al. (1998), Dunwoodie et al. (2002), Takahashi et al. (2003), Kusumi et al. (2004), Loomes et al. (2007), Chapman et al. (2011), Bochter et al. (2022)
*Jag1^−/−^*	Embryos die neonatally by E10	Normal somitogenesis with minor defects like butterfly vertebrae	NR										Lindsell et al., 1995, Xue et al. (1999)
*Notch1^−/−^*	Embryos die neonatally by E10	Disorganized and condensed PSM, delayed somitogenesis (form fewer somites by E10 vs. WT), somite lineages formed normally				NA; but expanded domain	CL except caudally	NA	NA at E8.5 *(* *in situ*); ∼2-fold DR (microarray)	DR			Swiatek et al. (1994), Conlon et al. (1995), Del Barco Barrantes et al. (1999), Leimeister et al. (2000), Loomes et al. (2007), Niwa et al. (2011)
*Notch2^−/−^*	Embryos die neonatally by E11.5	Normal somite formation	NR										Hamada et al. (1999), McCright et al. (2001)

NA, Not Affected; CL, complete loss; UR, Upregulated; DR, Downregulated; NR, not reported.

Despite this core function of *Hes7*, somites are formed nonetheless in *Hes7* ^*−/−*^ mice, albeit disfigured. This suggests the possibility of other core clock genes or the presence of signaling pathways like FGF or Wnt regulating the core clock circuit alongside Notch. Indeed, several Notch loss-of-function (LOF) mutant embryos that lose all cyclic *Hes7* activity in the rostral and medial E9.5-E10.5 PSM, still show some caudal *Hes7* expression, which is abrogated upon FGF inhibition (Ferjentsik et al., 2009). This suggests that FGF-induced *Hes7* expression in the posterior PSM acts independently of Notch and possibly acts as a clock initiator. Whether or not this FGF-driven *Hes7* is cyclic needs to be systemically characterized since one study has shown it to be non-cyclic (Ferjentsik et al., 2009), while another showed that this expression is dynamic (Niwa et al., 2007), despite both studies using *in situ* hybridization of the E9.5 caudal PSM. Furthermore, both *Dll1* ^*−/−*^ and *Notch1* ^*−/−*^ PSM at E9.5 show a caudally restricted *Hey2,* but not *Hey1* expression, strengthening the idea of a caudally restricted Notch-independent clock centered around bHLH-transcription factors (Leimeister et al., 2000). More evidence of a peripheral role of *Hey1* comes from the fact that its ubiquitous upregulated expression throughout the E9.5 PSM could still form up to ∼13 somites (Feller et al., 2008).

Surprisingly, the functioning of the core gene *Hes7* appears to be RBPjk-independent despite its known canonical requirement for Notch target gene expression (Figure 1). *RBPjk* ^*−/−*^ embryos, which die at E9.5, still show cyclic *Hes7* activity in the PSM (Ferjentsik et al., 2009). In agreement, a recent study used live light-sheet imaging of transgenic *Lfng* reporter carrying mice to study the onset of oscillatory dynamics in E6.5 mouse embryos and showed two interesting observations. First, *Lfng* shows a huge burst in its expression followed by two to three oscillations before the onset of somite formation. Second, and more importantly, *RBPjk* ^*−/−*^ embryos also show four to five *Lfng* oscillations prior to the onset of somitogenesis, but form disrupted somite borders subsequently (Falk et al., 2022). Although *Hes7* acts upstream of *Lfng* and as a result, this burst of *Lfng* expression does not inform us about *Hes7* dynamics, these results suggest an RBPJk-independent Notch activation. On the other hand, mice embryos mutant for *Psen1/2,* which lack γ-secretase activity do not form any somites (Huppert et al., 2005; Ferjentsik et al., 2009). These results could suggest a non-canonical Notch pathway downstream of Psen1 cleavage diverging before *RBPjk* function that still activates the core and peripheral clock genes to regulate the mouse segmentation clock.

### *her7* acts as the core clock gene during zebrafish segmentation

In zebrafish, due to the high number of gene duplications (Taylor et al., 2001), there are approximately 23 *her/hey* genes of which 10 are expressed in the PSM (Table 2). Of these, 7 genes-*her1, her7, her2, her4, her12, her15,* and *zhey1* oscillate (Table 2; Holley et al., 2000; Oates and Ho, 2002; Winkler et al., 2003; Sieger et al., 2004; Shankaran et al., 2007; Krol et al., 2011). One exception is *her6* which is expressed in the PSM only between the 3 and 5-somite stage embryo, following which its expression is undetectable in the PSM and restricted only to newly formed somites (Pasini et al., 2004). Each of these different *her* genes are critical for regulating segmentation, but to different extents.

Embryos at the 5–10 somite stages in which *her7* expression was disrupted either by morpholino-mediated knockdown or by its overexpression, resulted in a massive downregulation of its own transcription, that of *her1* and other clock genes like *deltaC, her12*, and *her15* (Oates and Ho, 2002; Gajewski et al., 2003; Giudicelli et al., 2007; Shankaran et al., 2007). Loss of *her7* transcription also resulted in disrupted somite borders and fused somites throughout the embryonic axis after a 5-somite delay (Henry et al., 2002; Giudicelli et al., 2007). Loss of *her1* expression by a genomic deletion, morpholino, or overexpression also resulted in a complete loss of its transcription, however, *her7* and other cyclic gene transcripts including *deltaC, her12*, and *her15* were attenuated but not fully lost, and were still cycling (Holley et al., 2002; Oates and Ho, 2002; Gajewski et al., 2003; Giudicelli et al., 2007; Shankaran et al., 2007). In agreement, neither *her1*-deletion nor *her1*-morpholino induced any severe segmentation phenotypes, other than deformed boundaries and elongated first few somites (Henry et al., 2002; Schröter and Oates, 2010). Furthermore, these defective boundaries were only observed in ∼85% of mutant embryos, suggesting incomplete penetrance which was not observed in *her7* mutant embryos (Keseroglu et al., 2023).

These loss-of-function phenotypes suggest that *her7* and *her1* may behave as a core and peripheral clock gene respectively (Figure 6B), despite the fact that the two genes are genomically paired, are co-expressed, and show overlapping expression patterns in the PSM (Gajewski et al., 2003; Zinani et al., 2020). In addition, a combined loss of *her1* and *her7* led to a higher penetrance of the somitic defects by disrupting somite borders along the whole embryonic axis including the first few somites indicating a complementary function or redundancy between the two genes (Henry et al., 2002; Oates and Ho, 2002; Sieger et al., 2004).

Unlike *her1* or *her7*, another bHLH-TF *her13.2* (*hes6* homolog) is independent of Delta-Notch signaling and under the direct control of FGF signaling. Due to this, *her13.2* does not oscillate in the PSM, but is expressed in a posterior-to-anterior gradient (Kawamura et al., 2005). Potentially as a result of this differential expression, a complete loss of *her13.2*, either by a genomic deletion or morpholino-mediated knockdown did not result in any severe segmentation phenotypes, and instead slowed down the clock, resulting in fewer (six to seven fewer), but bigger somites (Schröter and Oates, 2010). Interestingly, the addition of a *her1-*morpholino in a *her13.2* ^−/−^ background or alongside *her13.2*-morpholino resulted in severe disruption of segmentation along the entire embryonic axis, indicating a genetic interaction between the two genes (Schröter and Oates, 2010). Morpholino-mediated knockdown of either *her4* or *her6* alone resulted in a severe disruption of *her1* expression in the 10–12 somite stage embryo but showed minor segmentation defects with few somitic fusions (Takke and Campos-Ortega, 1999). This is particularly surprising given that *her6* is not expressed in the PSM at this stage, indicating interesting somite-PSM genetic crosstalk which has not yet been seen in mice or chick. Similar to *her13.2*, double morpholino-knockdown of *her1* and *her4* resulted in severe defects with multiple fused somites (Takke and Campos-Ortega, 1999).

Four other PSM-specific *her* genes, *her11*, *her12, her15*, and z*hey1* are only partially important for the maintenance of the segmentation clock. Misexpression of either *her12* or *her15* in the 10–12 somite stage embryos disrupted the cycling of clock genes and showed diffused somite borders in only ∼50% of the embryos (Shankaran et al., 2007). This low penetrance could be due to the wide range of homo/heterodimers that the Her proteins form to regulate gene expression (see below). An even lesser effect is seen when using MO-mediated knockdown of *her11* or z*hey1* at the 8–10 somite stage, where cyclic expression of *her1/7* and other cyclic genes like *deltaC* is unaffected (Sieger et al., 2004).

Despite these diverging roles of individual bHLH-transcription factors, these results provide evidence that the *hairy/Hes/her* genes control their own expression, as well as that of other oscillating bHLH genes and other clock genes in the segmentation clock (Figures 6A, B; Bessho et al., 2001a; Ferjentsik et al., 2009; Shifley et al., 2008; Zhang and Gridley, 1998). Finally, although *Lfng* is expressed in the segmented somites, it does not oscillate in the zebrafish PSM (Mayburd et al., 2001; Prince et al., 2001). This emphasizes that, at least in zebrafish and mice, *Hes7/her7* genes act as the core clock genes (Figure 6B).

### Lunatic fringe, but not bHLH TFs, is the core driver of chick segmentation

Expression studies in chick have also shown a similar functional divergence between bHLH genes like *c-hes1*, *c-hey1, c-hairy1, c-hey2, c-hairy2, and c-hes5,* all of which show different expression pattern in the chick PSM at HH stage 9–10 (Palmeirim et al., 1997; Jouve et al., 2000; Leimeister et al., 2000; Krol et al., 2011).

Contrary to mouse or zebrafish, in chick, *c-lfng* may act as a core player (Figure 6B). Overexpression of *c-lfng* leads to a downregulation and loss of *c-lfng, c-hairy1, and c-hairy2* cyclic mRNA expression in the PSM, which phenocopied Notch inhibition in the chick embryo (Dale et al., 2003). Additionally, microRNA-125a-5p-mediated post-transcriptional regulation of *c-lfng in vivo* is essential for chick segmentation suggesting that a tight control of its expression in the PSM is crucial for driving the segmentation clock (Riley et al., 2013). Interestingly, mir-125a-5p is dispensable for the mouse segmentation clock (Wahi et al., 2017), strengthening the view of a more core function of *c-lfng* in chick compared to the *Hes* genes in mice. In line with *c-lfng* being a core clock gene during chick somitogenesis, it also plays a critical role in somite polarization. In the anterior PSM, *c-lfng* is expressed as a tight band in the presumptive somite border. Mis-regulation of *c-lfng* expression results in disrupted somite borders and compartmentalization (Dale et al., 2003). Interestingly, when a group of PSM cells at the presumptive somite border were transplanted into an un-segmented PSM region, it induced ectopic fissure and border formation in the transplanted area (Sato et al., 2002). Essentially, border cells contained positional information about border formation which was encoded by a high *c-lfng* expression. This information was maintained even when transplanted elsewhere in the PSM resulting in ectopic somite borders.

A core function of *hes/hairy* genes in the chick segmentation clock cannot be ruled out; however, the lack of studies characterizing their roles through RNAi-mediated knockdowns makes it difficult to accurately predict these functions.

### Half-lives and transcriptional delays of bHLH-Transcription factors

There are several features of the mouse segmentation clock that give it its characteristic 120-min period, one of them being delays associated with transcription and protein expression (Jensen et al., 2003; Monk, 2003; Hirata et al., 2004). One of the events that can affect these delays is splicing (Takashima et al., 2011; Alpert et al., 2017).

Introduction of an intronless *Hes7* transgene fused to a luciferase showed that the luminescence preceded the endogenous protein expression domain by ∼21 min in E10.5 mouse PSM explants, suggesting a reduced delay in protein expression (Takashima et al., 2011). Surprisingly however, the period of the transgenic *Hes7* did not significantly change, possibly due to interference from the endogenous *Hes7*. Mutant mice containing intronless *Hes7* form highly disfigured, fused somites and a truncated body axis phenocopying *Hes7* ^*−/−*^ mice, indicating the importance of fine delays in maintaining correct patterning (Takashima et al., 2011). In fact, transgenic mice containing intronless *Hes7*, and not WT *Hes7* showed severe segmentation defects resembling *Hes7* ^*−/−*^ mice (Harima et al., 2013). This suggests that intronic delay, but not copy number *per se* is essential to maintain proper *Hes7* oscillations, and thereby somitogenesis. In contrast, another study reported kinked tails in *Hes7* ^*+/−*^ mice, indicating that the dose of Hes7, only when it drops below WT levels, could be important for somitogenesis (Bessho et al., 2001b).

Remarkably, introducing a transgene lacking two of the three *Hes7* introns did not lead to any severe segmentation defects, but formed additional cervical and upper-thoracic segments, suggesting an acceleration of the clock. Furthermore, the removal of any one intron did not affect the clock or somitogenesis at all (Harima et al., 2013). These results show that while intronic delay is crucial, this criticality is dependent upon the number of introns present in a “dose-dependent” manner.

Once the *Hes7* mRNA is spliced and the Hes7 protein is made, it needs to be degraded with rapid turnover to allow for the next cycle of expression to begin. Stabilizing the mouse Hes7 protein by mutating ubiquitination sites on Hes7 prevented its degradation and increased its half-life from 22 min (WT) to ∼30 min when measured *in vitro* (Hirata et al., 2004). *In silico* models showed that this small increase in Hes7 half-life severely dampened oscillations after 3-4 cycles. In agreement, knock-in mice containing this mutant *Hes7* showed normal somitogenesis and cyclic *Hes7* and *Lfng* expression until the 3-4 somite stage, following which the clock was fully disrupted resulting in defective segmentation (Hirata et al., 2004). These results show that protein turnover rates are central to maintaining proper cyclic gene expression, and therefore proper body segmentation.

In fact, a recent study used a transgenic *Hes7* construct to show that the mouse Hes7 has an ∼2-fold faster decay rate compared to the human HES7, which correlates with the ∼2-fold faster mouse clock (Matsuda et al., 2020a). The authors also found that the human HES7 decayed at a mouse-specific rate when the transgene was expressed in the mouse PSM *in vitro*. Global translation inhibition in Zebrafish embryos at the 12–14-somite stage revealed that the half-life of Her7 is only ∼3.5 min (Giudicelli et al., 2007; Ay et al., 2013), compared to an ∼20 min and ∼40 min half-life of mouse Hes7 and human HES7 respectively (Matsuda et al., 2020a). These Hes/Her protein half-lives correlate with species-specific clock periods. These studies suggest that beyond the gene sequence itself, the surrounding transcriptional, translational, and degradation machinery plays a crucial role in regulating species-specific developmental timing.

However, this correlation may not extend to the chick segmentation clock, where blocking *de-novo* protein synthesis did not affect the dynamicity of *either c-hairy1 or c-hey2* expression. This suggests a different mechanism for cyclic expression in chick (Palmeirim et al., 1997; McGrew et al., 1998; Leimeister et al., 2000). As a result, transcriptional/protein decay rates may only be one of the determinants of species-specific clock periods, and further comparative studies will be necessary to uncover the other ones.

### Dimerization of bHLH-transcription factors

bHLH-TFs like the *Hes/her* genes have the ability to form homo/hetero-dimerize with other bHLH genes via their helix-loop-helix structures (Jones, 2004). Altering dimerization parameters of *Hes7 in silico* can alter clock properties in the mouse PSM, however *in vivo* data for the same is lacking (Song et al., 2011).

In zebrafish however, the dimerization network of bHLH-TFs has been more extensively characterized. *her* genes form homo- and hetero-dimers in a “dimer cloud” (Figure 6A; Schröter et al., 2012), and this dimerization ability is essential for pattern formation in the fish embryo (Henry et al., 2002; Zinani et al., 2020; Zinani et al., 2022). *her1* and *her7* are present head-to-head on the same chromosome which enables their co-transcription and co-repression by a Her1/7 dimer. One study mutated the *her1* and *her7* genes on opposite sister chromatids resulting in reduced correlated gene expression dynamics and disrupted somitogenesis (Zinani et al., 2020). Her7 and Her13.2 also form heterodimers and enable autorepression of the *her1* and *her7*, but also modulate the dimer topology within each cell crucial for the functioning of the clock (Figure 6A; Schröter et al., 2012). Her13.2 also affects the stability of the Her1/7 dimer via protein-protein competition and its loss results in increased stability of this dimer and a slower clock (Schröter et al., 2012). On the other hand, another study reported that Her7 acts as the critical node of the dimer topology and limits the availability of Hes13.2 to dimerize with the other cyclic Her proteins like Her1, Her12, and Her15 (Trofka et al., 2012). Indeed, electrophoretic mobility shift assays (EMSA) have shown that the *cis*-regulatory elements of *her1* contain Her12 binding sites (Brend and Holley, 2009), and that Her12 plays an important role in regulating *her1* expression (Shankaran et al., 2007).

Nonetheless, these studies suggest the complex interplay between bHLH-TFs necessary for somitogenesis, a feature not as extensive in mice or chick due to the limited number of *Hes/Hey* genes in the two species. This brings out an intriguing question- Does an extensive network of bHLH-TFs display redundancy in a way that makes zebrafish somitogenesis more robust to environmental/genetic aberrations? *her1* knockout embryos show only ∼85% penetrance in segmentation defects suggesting some functional compensation by other *her* genes (Keseroglu et al., 2023). More comparative studies using different *her* mutants and across different species are necessary to address this question.

Upstream players of the Notch signaling pathway like the ligands and receptors play an important role in regulating and synchronizing this complex transcriptional landscape painted by bHLH-TFs, and the loss of these upstream components also disrupts somitogenesis.

## Species-specific divergent functions of Notch ligands and receptors during segmentation

### *Dll1* has a stronger influence on mammalian development than *Dll3*

The mouse PSM expresses three ligands- *Dll1, Dll3*, *Jag1,* and two Notch receptors- *Notch1 and Notch2* (Swiatek et al., 1994; Lindsell et al., 1995; Shawber et al., 1996; De Angelis et al., 1997; Dunwoodie et al., 1997; Hamada et al., 1999). These different receptors and ligands play distinct roles during somitogenesis. While *Dll1* and *Dll3* null embryos show defects in somitogenesis, *Jag1^−/−^* embryos do not, except for minor defects like butterfly vertebrae (Table 3). Phenotypes of *Dll1^−/−^* and *Dll3* ^−/−^ embryos suggest that the expression of clock genes is independently regulated in the rostral and caudal PSM, and while Dll1 is required in both, Dll3 is only required in the caudal PSM (Del Barco Barrantes et al., 1999; Jouve et al., 2000; Dunwoodie et al., 2002). For example*, Dll1^−/−^* mice show a complete loss of *Lfng* in the E8.5 mouse PSM whereas *Dll3^−/−^* E9.5 embryos only lose the caudal *Lfng* expression (Del Barco Barrantes et al., 1999; Kusumi et al., 2004). This is also consistent with another study showing that the loss of *Dll3* is epistatic to the loss of *Lfng* in E10.5 embryos (Riley et al., 2013; Bochter et al., 2022).

Furthermore, while *Dll1^−/−^* E9.5 embryos lose all *Hes7* expression except in the caudal PSM, *Dll3^−/−^* E9.5 embryos still show normal cyclic *Hes7* expression (Kusumi et al., 2004; Niwa et al., 2007; Sewell et al., 2009). Two other studies have challenged this by also using *in situ* hybridization showing that while *Dll3^−/−^* E9 PSM do not lose any *Hes7* expression, they still become non-oscillatory (Chen et al., 2005; Bochter et al., 2022). On the other hand, *Dll1^−/−^* and *Dll3^−/−^* embryos both show complete loss of *Hes1* and *Hes5* expression (Del Barco Barrantes et al., 1999; Jouve et al., 2000; Dunwoodie et al., 2002).

These data suggest while Dll1 is crucial for regulating the expression of all *Hes* genes and *Lfng*, Dll3 is crucial only for *Lfng* and perhaps more peripheral bHLH gene expression. Potentially due to this “stronger” influence of *Dll1*, *Dll1^−/−^* mice are unable to establish any rostrocaudal compartmentalization, fail to make epithelial somites, show incomplete axis formation, and eventually die by E12 (De Angelis et al., 1997; Preuße et al., 2015; Shimojo et al., 2016). On the other hand, loss of *Dll3* results in less severe phenotypes where although somite borders are not visible, clear metameric units are evident, albeit highly disorganized, which eventually also differentiate into different somitic lineages (Dunwoodie et al., 2002). Furthermore, *Dll3^−/−^* mice die postnatally within 10 days of birth (Dunwoodie et al., 2002). These phenotypes also reinforce the influence of a loss of core clock genes in *Dll1^−/−^* embryos *versus* the loss of more peripheral genes in *Dll3* ^−/−^ embryos. Interestingly, there are many more documented human *DLL3* clinical mutations (*versus DLL1*) that result in a disorganized vertebral column. To date, there have been 28 reported *DLL3* mutations resulting in a form of spondylocostal dysostosis (SCDO), SCDO1, which is a type of vertebral malformation (Kusumi et al., 2004; Umair et al., 2022). On the other hand, there has been only 1 *DLL1* mutation causing another type of SCDO, SCDO7 (Barhoumi et al., 2019). One plausible reason for this could be that, similar to mice, mutations in *DLL1* (as opposed to *DLL3*) are likely to be more embryonic lethal in humans, resulting in this disparity in reported cases.

Both *Dll1^−/−^* and *Notch1^−/−^* E8.5-E9.5 PSM completely lose *Hey*2 expression except in the caudal PSM (Leimeister et al., 2000; Niwa et al., 2011), but whereas *Dll1^−/−^* mice show a complete loss of *Hey1*, it is unaffected in *Notch1^−/−^* embryos (Table 3; Leimeister et al., 2000). This suggests that for *Hey2* expression, Dll1 and Notch1 act linearly, but there may be additional ligand-receptor pairs regulating *Hey1* expression in the mouse PSM. Additionally, while *Dll1^−/−^* PSM lose all *Lfng* expression and somite polarity, *Notch1^−/−^* PSM show only a reduced *Lfng* expressed with diffused borders (Leimeister et al., 2000). Somite formation is retarded in these embryos which, by E9.5, have only formed 14–16 somitic pairs compared to the 20–25 pairs in WT embryos, and eventually die by E11-11.5 (Swiatek et al., 1994; Conlon et al., 1995). The fact that *Notch1^−/−^* embryos can form somites is surprising given that *Notch2* ^−/−^ embryos also do not show any somitogenesis defects, but *Psen1/2 ^−/−^* embryos show an absence of somites altogether (Hamada et al., 1999; Huppert et al., 2005; Ferjentsik et al., 2009). Notch1 likely serves as the core receptor during somitogenesis. In *Notch1^−/−^* embryos, partial compensation by *Notch2* allows the retarded formation of 14–16 somites before embryonic death. On the other hand, when *Notch2* is knocked out, *Notch1* can fully compensate for its function. Alternatively, *Psen1/2* may possess a Notch-independent role in regulating somitogenesis.

All of the above studies combined show that a complex, spatiotemporally regulated medley of Notch ligands, receptors, and their targets is of critical importance in regulating the mouse segmentation clock.

### Zebrafish *deltaC* may be more similar to the mouse *Dll1*

In zebrafish, *deltaC* (*beamter; bea*) and *deltaD* (*after eight; aei*) LOF mutant embryos show different segmentation phenotypes in the PSM, suggesting that they may serve separate roles in regulating the segmentation clock (Holley et al., 2000; Holley et al., 2002; Mara et al., 2007). While *deltaD* (*aei*) mutants display defective somitogenesis after 8-9 somites, *deltaC* (*bea*) mutants show disruption following just 3-4 somites indicating that, like *dll1*, *deltaC* acts as a more core regulator of segmentation (Van Eeden et al., 1996; 1998; Jiang et al., 2000). In fact, *aei/bea* double mutants show the same segmentation phenotypes as the *bea* mutants, reflecting the more primary function of *deltaC* (Jülich et al., 2005). This is in contrast to expression patterns of cyclic genes, where *in situ* hybridization in *bea* mutants display a salt-and-pepper expression of *her1* and *her7* throughout the 8–12 somite stage PSM, whereas *her1* expression is completely lost (except in the anterior and posterior tips) in *aei* mutants (Holley et al., 2000; 2002). This phenotype is partially reminiscent of *Hes7* expression in *Dll1* ^*−/−*^ mouse PSM. In contradiction, one study used high-resolution single-cell imaging and fluorescent *in situ* hybridization to show that *deltaD* ^*−/−*^ embryos show no posterior expression of *her1* in the 12-somite stage PSM (Mara et al., 2007). As a result, it may be difficult to directly associate ligand similarities between different species based on mutant phenotypes alone. Nonetheless, these studies suggest a more “synchronization" role for DeltaC such that its loss results in a random, salt-and-pepper expression, but a more “driver” role for DeltaD such that its loss results in a complete loss of cyclic gene expression (Holley et al., 2002).

**FIGURE 7:**
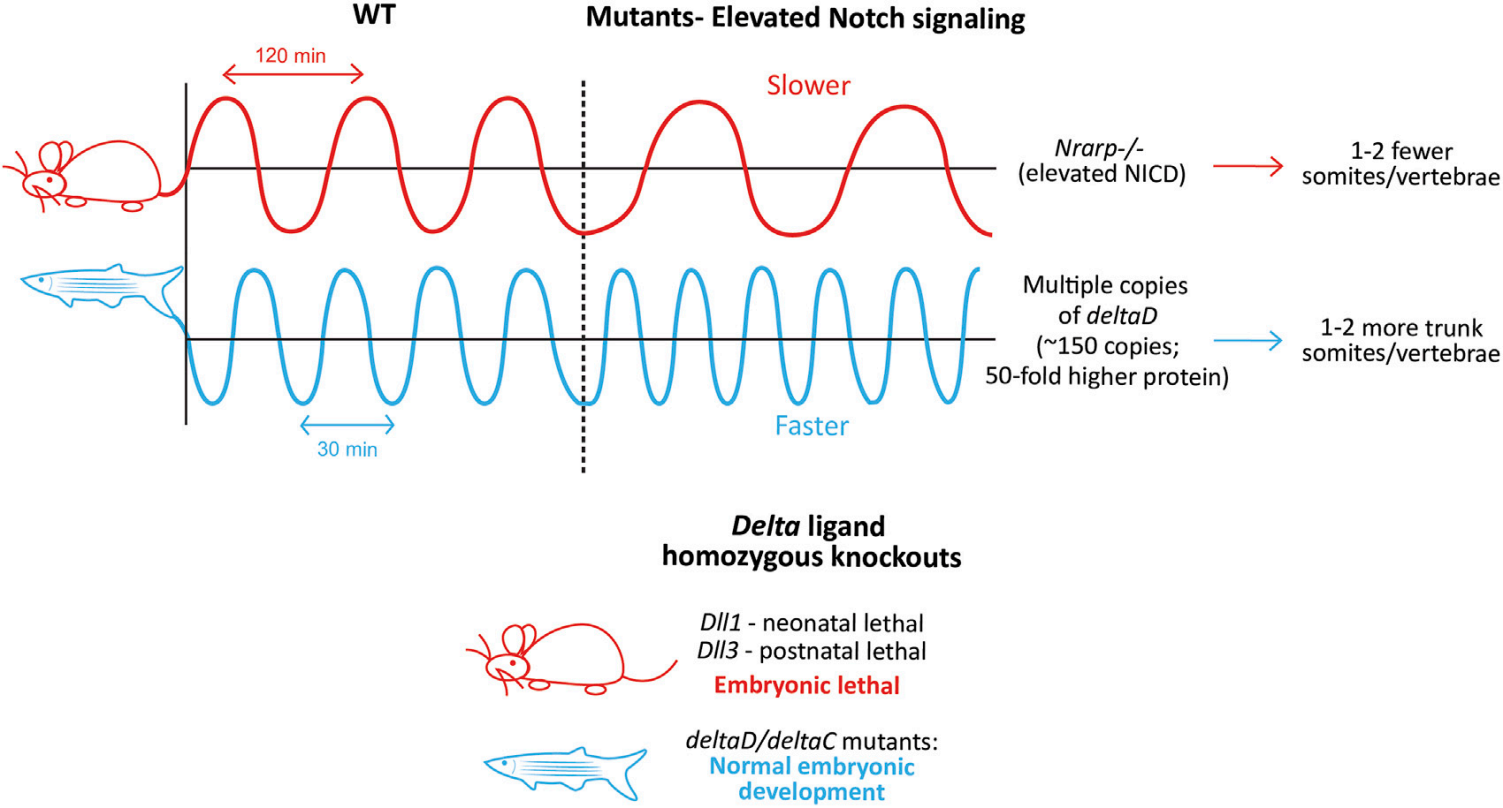
Species-specific differences in clock dynamics and Notch mutant phenotypes. Elevated levels of the Notch signaling pathway opposingly affect the mouse and zebrafish clock periods, and as a result, the final number of somites/vertebrae formed (Upper panel). Species-specific differences in the requirement of Delta ligands during embryonic development. Mouse-red; zebrafish-blue (Lower panel)..

Irrespective of the ligand or receptor function lost, at least a few somite cycles must elapse before the blockade of Notch signaling and severe segmentation defects. This can happen if Notch signaling regulates synchronization primarily, but not the clock directly. Alternatively, it is also possible that Notch signaling in fish is important only for the posterior clock, but not the anterior one, where a different signaling pathway is a primary driver. In fact, a recent study showed that *her1* ^*−/−*^ *;her7* ^*−/−*^ fish can still show a proper segmentation profile if provided with exogenous pulses of FGF/Erk signaling inhibition between the 17–22 somite stage (Simsek et al., 2023). These results are complemented by the observation that *deltaD;deltaC* single and double knockout fish embryos show normal axial development, and are viable and fertile (Jülich et al., 2005), as opposed to ligand knockout mice which are embryonic lethal (Figure 7, Lower panel).

## The levels of Notch signaling affect the segmentation clock properties

### An increase in Notch signaling levels slows down the mouse clock

While the presence of Notch signaling is necessary for the integrity of the mouse clock, the level of Notch signaling itself can also regulate the clock by changing its properties (Figure 7, Upper panel). For example, mice with a LOF mutation in the Notch regulated ankyrin repeat protein (*Nrarp*), a cyclic Notch target that represses NICD cleavage in the mouse PSM (Sewell et al., 2009), form ∼2 fewer vertebrae compared to WT mice, and ∼50% of homozygous mutant mice show a kinked tail (Kim et al., 2011). In line with the function of *Nrarp*, *Nrarp* ^*−/−*^ E10.5 PSM show ∼1.9-fold higher NICD levels. Furthermore, when WT pregnant mice were treated with LY-411575 (0.1 mg/kg; a γ-secretase inhibitor that inhibits Notch signaling) every 24 h starting at E7.5, NICD levels in E10.5 embryos dropped by 10%–20%, resulting in a slightly faster clock in ∼50% embryos that formed 1 extra somite compared to the untreated controls. Remarkably, the same dose of LY-411575 treatment to mice carrying *Nrarp* ^*−/−*^ embryos also resulted in a rescue in ∼50% of embryos forming one additional somite compared to the untreated mutants. This suggests that the loss of *Nrarp* results in an increase in NICD levels, in turn increasing the period. Interestingly however, the cyclic expression of clock genes like *Hes7, Lfng*, and that of NICD were unaffected in these mutant embryos. As a result, *Nrarp* may also be a more peripheral component, consistent with studies showing that the loss of *Dll3* results in the complete loss of *Nrarp* expression (Sewell et al., 2009; Bochter et al., 2022). One plausible explanation for the increased period in *Nrarp* ^*−/−*^ embryos is that higher levels of Lfng may be necessary to build up to effectively repress the higher amount of NICD, which may take significantly more time, hence slowing the clock. However, increasing the half-life of NICD also slowed the clock (Wiedermann et al., 2015). This indicates that not just the levels of NICD, but the time spent by NICD in the cell also affects clock periodicity, suggesting a Lfng-independent mechanism.

### An increase in Notch signaling levels speeds up the fish clock

The fish clock is also sensitive to the levels of Notch signaling, however in the direction opposite to that of mice. Transgenic zebrafish embryos containing multiple copies of *deltaD* produced more somites and vertebrae compared to WT embryos (Figure 7, Upper panel; Liao et al., 2016). Interestingly, the authors generate two lines, *Dover* with ∼10-fold higher DeltaD levels in the homozygotes (copy number ∼15), and *Damascus* with ∼50-fold higher DeltaD in the heterozygotes (copy number ∼150). While both the homozygous and heterozygous *Dover* mutants showed normal somitogenesis, none of the homozygous *Damascus* mutants survive. Surprisingly, the heterozygous *Damascus* mutants develop normally, and form an overall 2 additional trunk somites compared to WT siblings, with clock periods being ∼1.5 min faster specifically in the trunk region (4–19 somite stage). In contrast, neurogenesis was affected even with the slightest increase in DeltaD levels, suggesting the robustness of somitogenesis to tolerate gene expression changes compared to other lineages.

On the other hand, DAPT treatment (another γ-secretase inhibitor that inhibits Notch signaling) or homozygous *aei*/*des* embryos show a slower segmentation clock at the 3-4 somite stage (heterozygotes do not, even though protein levels are halved (Liao et al., 2016)), resulting in larger anterior somites before the onset of segmental defects (Herrgen et al., 2010). In the presence of DAPT, *Damascus* mutants showed no change in the clock period (Liao et al., 2016). This suggests that while no *deltaD* copies (*aei* embryos) confer segmental defects, moderately abundant copies do not affect somitogenesis, or even embryonic development. This could be because *deltaD* does not oscillate in the fish PSM, and as a result, too much of it does not disrupt its endogenous expression dynamics. Overexpression of *Dll1* in mouse embryos can disrupt *Dll1* oscillations and thereby somitogenesis (Shimojo et al., 2016). As a result, it is possible that the introduction of multiple copies of *deltaC* disrupts somitogenesis.

These results reinforce that while in mice, Notch signaling primarily drives the expression of oscillatory genes, in zebrafish, Notch signaling only synchronizes and propagates *her1/7* oscillations. This is supported by the ‘delayed coupling theory’ where reduced Notch signaling results in weaker coupling strength and higher delays in information transfer between neighboring PSM cells, thereby resulting in a collective slower clock (Morelli et al., 2009; Herrgen et al., 2010; Liao et al., 2016; Yoshioka-Kobayashi et al., 2020). Due to the lack of genetic tools, similar Notch signaling manipulative studies have not been reported yet in chick embryos. It would be interesting to characterize the effects of *in ovo* electroporation of *c-delta1* or *c-notch1*/c-NICD cDNA on the chick segmentation clock time.

## Notch signaling and cell-cell synchronization

### Lfng acts as a synchronizer in the mouse PSM

The role of Notch signaling as a synchronizer in mouse embryos has evolved over many decades, since initially Notch signaling was only considered to be essential to maintain gene oscillations in individual PSM cells, based on Notch ligand and receptor LOF mutant phenotypes. Two studies showed that when mouse E10.5 or chick PSM tissue (14–18 somite stage) is dissected into multiple fragments, they are each able to oscillate maintaining the correct clock schedule compared to an intact tissue (Maroto et al., 2005; Masamizu et al., 2006). This showed that at least at the global level, groups of cells maintained synchrony despite being cultured separately. With the advent of single-cell resolution imaging, the role of Notch signaling as a cell synchronizer alongside being a ‘switch’ to maintain oscillations became easier to study. Masamizu et al., 2006 showed first that dissociated E9.5-10.5 transgenic mouse tailbud cells showed asynchronous and unstable *Hes1* oscillations *in vitro* (Masamizu et al., 2006). Another study culturing dissociated E9.5 transgenic mouse tail bud explants showed similar dampened and weak *Lfng* oscillations (Hubaud et al., 2017). A similar experiment in 14–18 somite stage chick embryos showed that when the PSM was dissociated and cultured *in vitro* in separate pools, c-Lfng oscillated asynchronously across pools as assayed by *in situ* hybridization, suggesting that cell-cell communication is necessary to maintain stable, synchronous oscillations (Maroto et al., 2005). However, was this property Notch-dependent? Using an *in vitro* culture technique of mouse E10.5 tail bud explants, Tsiairis and Aulehla, (2016) showed that DAPT treatment affected the ability of dissociated and reassembled PSM cells to self-organize into spatiotemporal wave patterns upon reaggregation *in vitro* (Lauschke et al., 2012; Tsiairis and Aulehla, 2016).

A major obstacle in understanding the role of Notch as a synchronizer in mouse segmentation stems from the fact that Notch signaling directly controls *Hes7* or *Lfng* oscillations throughout the PSM. As a result, the dissociation of the role of Notch as a driver of oscillations and as a synchronizer between oscillators is a challenge. The dampening and instability of *Hes7* and *Lfng* oscillations in Notch pathway LOF mutants or DAPT-treated embryos may be because of reduced expression of these genes rather than due to a loss of synchrony. To combat this, one study used chimeric mouse embryos consisting of WT and *Dll1* ^*−/−*^ cells and showed that the synchronization rates in the E10.5 PSM reduced linearly with decreasing number of WT cells in the chimeras (Okubo et al., 2012). They also used chimeric mice containing WT and *Lfng* ^*−/−*^ cells to show that Lfng could regulate *Hes7* oscillations non-cell autonomously. Another study also used immortalized cell lines to show that Lfng can repress *Dll1* (alongside NICD) cell-autonomously to regulate levels of Notch signaling cell-autonomously (via NICD) and non-autonomously (via Dll1) (Okubo et al., 2012; Bochter et al., 2022). These data suggest a unique role of Lfng in being responsible for maintaining synchronization during somitogenesis (Figure 6A). A recent study supports the these results through single-cell tracking of Hes7-positive and negative cells in *Lfng* ^*−/−*^ PSM that showed individual oscillators lose synchrony (Yoshioka-Kobayashi et al., 2020).

### Her1/7 directly *repress deltaC* in zebrafish

In zebrafish Notch-LOF embryos, the first few somites develop normally and the progressive worsening of somitogenesis has been attributed to a loss of synchronization between individual oscillators. For example, in a ‘re-synchronization assay’, zebrafish embryos treated with DAPT that start to form disfigured somites after about 5 somites, promptly re-form normal somites after DAPT withdrawal due to the “resynchronization” of the clock (Liao et al., 2016; Uriu et al., 2021). As the tissue recovers following DAPT withdrawal, the defective and normal somites intermingle transiently showing that recovery occurs at multiple levels: At the cellular level with rapid resynchronization between individual cells via Delta-Notch signaling; and the tissue level with the transfer of this resynchronization “information” throughout the PSM (Uriu et al., 2021). *deltaD*/*aei* or *notch1/des* heterozygous mutants fail to resynchronize effectively after DAPT treatment, whereas *deltaC/bea* mutants recover similarly to WT embryos, suggesting ligand-specific roles in carrying out synchronization (Mara et al., 2007). In contrast, embryos with multiple *deltaD* copies can resynchronize faster than their WT siblings (Liao et al., 2016).

Similar to mice, advances in single-cell resolution imaging of Her1 oscillations *in vivo* showed that individual cells oscillate autonomously, which begin cycling out of phase in *deltaD (aie), deltaC (beamter)*, and *notch1a (des)* embryos (Delaune et al., 2012). A more recent study corroborated this by culturing the caudal PSM cells *in vitro* at low densities and detecting cell-autonomous, but asynchronous and noisy *her1* oscillations (Webb et al., 2016). This high background noise was absent in cells belonging to an intact PSM, suggesting a role for collective tissue-level processes in increasing the precision of the clock.

Interestingly, the suggested mechanism of synchronization in zebrafish is slightly different from mice (Figure 6A). The Her1/7 dimer is predicted to directly repress *deltaC* expression levels (Horikawa et al., 2006). One study showed that the *deltaC* expression pattern in *her1*-LOF fish is only altered in the anterior PSM but not posterior, suggesting that this predicted mechanism of synchronization differs along the embryonic axis (Choorapoikayil et al., 2012). However, this remains to be systematically characterized.

## Insights from non-classical model systems and future perspectives

Much of the early knowledge about body segmentation before studies in chick embryos came from arthropods like *Drosophila* (Davis and Patel, 1999; Pourquie, 2003). However, unlike vertebrates, genetic studies have shown that Notch signaling does not have a link to fly segmentation (Johnston and Nüsslein-Volhard, 1992). Surprisingly, homologs of the genes *delta, notch*, and *hairy* all show a segmental expression pattern during early development of another arthropod, the spider *Cupiennius salei* (Stollewerk et al., 2003). RNAi-mediated knockdown of *delta* and *notch* resulted in disorganized *hairy* segmentation, similar to vertebrates. Similar segmented expression of Notch components and their roles in embryonic segmentation have been observed in other invertebrates like the cockroach *Periplanata americana* and the centipede *Strigamia maritima,* but not in the cricket *Gryllus bimaculatus* (Pueyo et al., 2008; Reviewed extensively in; Kainz et al., 2011; Brena and Akam, 2013; Clark et al., 2019). This suggests that a wider sampling of invertebrate species beyond classical models like *Drosophila* can reveal otherwise unidentified complex ancestral segmentation mechanisms. As a result, it is essential to expand our horizon on this developmental process by using non-classical *in vivo* and *in vitro* model systems, including other non-model vertebrates and potentially even invertebrate species to better understand this process.

### Reptiles

Corn snake embryos form 200–300 somites with a timing of ∼100 min per somite (Table 4; Gomez et al., 2008). Interestingly, the house snake which has very similar developmental characteristics to the corn snake, displays a clock of ∼60 min suggesting that small genetic differences within a given genus can drastically affect clock timing (Gomez et al., 2008). This correlation is challenging to verify when only using classical models like mouse and fish, which inherently are very different. Reptiles like the green anole lizard and alligator have also been used to study somitogenesis which unlike traditional model species, do not express *Lfng* in the PSM. While the corn snake does, it exhibits significantly more stripes in the PSM compared to mice, suggesting a different mode of clock gene regulation (Gomez et al., 2008; Eckalbar et al., 2012). In contrast, similar to classical models, *Dll1* displays oscillatory expression in the anole lizard (Eckalbar et al., 2012). Another example is *Dll3*, while expressed uniformly throughout the mouse PSM, is confined to the caudal-most PSM and displays static anterior bands in the anole lizard. These observations suggest possibly conserved and divergent mechanisms regulating the core *versus* peripheral clock players respectively in the reptilian segmentation clock.

**TABLE 4:** Species-specific segmentation clock periods across various *in vivo* and *in vitro* model systems.

Species	Segmentation clock period	References
*In vivo* PSM models		
Zebrafish	18–55 min	Stickney et al. (2000), Schröter et al. (2008)
Medaka	60–120 min	Elmasri et al. (2004), Vibe (2021)
House snake	60 min	Gomez et al. (2008)
Corn Snake	100 min	Gomez et al. (2008)
Chick	90 min	Stern et al. (1988), Pourquié (2004)
Mouse	120–180 min	Aulehla et al. (2008)
*In vitro* PSM models		
Rabbit	∼150 min	Lázaro et al. (2023)
Cattle	∼230 min	
Rhinoceros	∼230 min	
Marmoset	∼400 min	
Pig	∼226 min	Conrad et al. (2023)
Human	∼310 min	Orts-Llorca (1981), Chu et al. (2019), Matsuda et al. (2020a), Diaz-Cuadros et al. (2020)

### Other teleosts

Studies on other teleost fish species like *Oryzias latipes* (Medaka) and *Tagifuku rubripes* (Japanese pufferfish) also show some interesting clock divergence from zebrafish (*Danio rerio*) (Elmasri et al., 2004; Gajewski et al., 2006). Medaka embryos show a clock timing of ∼60 min at 26°C, whereas zebrafish embryos tick approximately two-fold faster at 25–30 min at 26°C suggesting similar intra-species differences like snakes (Table 4; Elmasri et al., 2004; Schröter et al., 2008). In fact, 60 min is the clock speed at the posterior PSM which slows down by almost half to ∼120 min in the anterior PSM of a 3-5 somite-stage medaka embryo (Vibe, 2021). Given that the anterior speed of the medaka embryo (unlike zebrafish embryos) is very similar to the posterior PSM clock in mice, understanding why a period gradient arises in medaka could help decode the underlying mechanisms of species-specific clock times. Genetic knockout phenotypes have also shown that the Medaka *her7* is functionally similar to the zebrafish *her1* and *her7* combined, and the Medaka *her1/11* to the zebrafish *her11* over *her1* (Elmasri et al., 2004; Sieger et al., 2006). These phenotypes further support the idea that teleost embryos use *her7* as a core clock driver and *her1/11* genes as more peripheral ones.

Another advantage that Medaka has for decoding the genetics underlying species-specific clock times is that different inbred Medaka sub-species show different *her7* clock periods (Seleit et al., 2023). They also have variable PSM and somite lengths, which positively correlate with clock periodicity. Crossbreeding species with different periods provides a useful approach to understand the genetic basis of clock periodicity. Accordingly, intercrossing these different Medaka species results in a wide range of meiotic recombination across the genome, yielding F2 progeny showing a wide range of clock periods (Seleit et al., 2023). The authors further show that while clock period scales with PSM size in parental fishes, these two parameters do not correlate in F2 progeny. On the other hand, somite size still scales with PSM size. This raises the possibility that there is a spectrum of somite numbers formed in the F2 embryos. However, this has not been shown. In addition, whole genome sequencing of F2 embryos revealed that the Medaka *dll1* is responsible for controlling PSM size, but not somite number/border formation or clock period. This result is in contrast to *Dll1^−/−^* mice where no somite borders are formed, indicating a potential evolutionarily divergent role of Notch ligands.

One way to combat the inability to perform inter-species crosses in mammals is to carry out *in vivo* or *in vitro* gene-swapping experiments. One study showed that the species-specific oscillatory period of *Hes7* is not entirely regulated by local genomic sequences (Matsuda et al., 2020a). In particular, the human segmentation clock ticks with a periodicity of ∼5 h (Chu et al., 2019; Matsuda et al., 2020b; Diaz-Cuadros et al., 2020) and when the mouse *Hes7* locus was swapped with the human *HES7 in vivo*, it results in only an ∼20–30 min extension of the mouse period when measured in E10.5 mouse PSM explants (Matsuda et al., 2020a). This genomic manipulation caused minor, but significant segmentation defects in the form of a curved spine and a kinked short tail. Possibly, mutant embryos containing the human *HES7* that showed a change in the clock time of >20–30 min may have been embryonic lethal, thereby only the embryos with the minimal clock perturbation survive. Furthermore, because this gene swapping was limited to a BAC construct extending only up to the 3′ UTR of the neighboring gene *Per1*, one cannot exclude the possibility of *cis*-elements downstream of *Hes7* affecting its periodicity. While there are only limited reports on how the segmentation clock and the circadian clock could interact genetically (Umair et al., 2022), the *PER1* gene also displays an in-phase gene oscillation phenotype with *HES7* in human PSM cells *in vitro* (Matsuda et al., 2020b).

### *In vitro* 2D and 3D systems

Aligning with the ideology of the importance of comparative developmental biology, recent studies have established embryonic stem cells or induced pluripotent stem cells (iPSCs) derived from various species of different sizes and gestational lengths such as marmosets, rhinoceros, cattle, rabbits (Lázaro et al., 2023) and pigs (Conrad et al., 2023) (Table 4). These species-specific PSCs display intrinsic developmental timing phenotypes when differentiated to a PSM and other lineages (Matsuda et al., 2020a; Rayon et al., 2020; Lázaro et al., 2023). The *in vitro* clock period observed across these different species also correlates with *in vivo* gestation times, body size, and biochemical reaction rates *in vitro* (Matsuda et al., 2020a; Conrad et al., 2023; Diaz-Cuadros et al., 2023; Lázaro et al., 2023). Therefore, carefully curated *in vivo* embryonic staging data can help determine how closely these *in vitro* systems can mimic somitogenesis. In addition, recent studies have showcased the power of both 2D and 3D mouse and human *in vitro* models in recapitulating hallmarks of somite formation, thereby giving us insights, especially into human development (Diaz-Cuadros and Pourquie, 2021; van den Brink et al., 2020; Budjan et al., 2022; Chu et al., 2019; Diaz-Cuadros et al., 2020; Matsuda et al., 2020b; Miao et al., 2022; Sanaki-Matsumiya et al., 2022; Veenvliet et al., 2020; Yamanaka et al., 2022). Multiple studies have shown human pluripotent stem cells (PSCs)-derived PSM cells capable of undergoing 3D morphological somite-like budding that can further our knowledge of how temporal information translates to spatial information in our species (Miao et al., 2022; Sanaki-Matsumiya et al., 2022; Yamanaka et al., 2022).

## Conclusion

In this review, we summarized existing knowledge on the segmentation clock biology by leveraging the similarities and differences of the mouse segmentation clock with that of zebrafish and chick. Through insightful comparisons with non-classical model organisms and novel *in vitro* models, we illuminate the profound importance of these systems in advancing our understanding of the segmentation clock and its evolution. In the future, we anticipate that novel *in vivo* and *in vitro* approaches will provide complimentary knowledge to better understand vertebrate segmentation and embryonic development.
